# Does residential mobility during pregnancy induce exposure misclassification for air pollution?

**DOI:** 10.1186/s12940-018-0416-8

**Published:** 2018-10-19

**Authors:** Olivier Blanchard, Séverine Deguen, Wahida Kihal-Talantikite, Romain François, Denis Zmirou-Navier

**Affiliations:** 1 Univ Rennes, EHESP, Inserm, Irset (Institut de recherche en santé, environnement et travail) - UMR_S 1085, F-35000 Rennes, France; 2 EHESP, Inserm, IPLESP (Institut Pierre Louis d’Epidémiologie et de Santé Publique) - UMR_S 1136, F-35000 Rennes, France; 3 LIVE UMR 7362 CNRS (Laboratoire Image Ville Environnement), University of Strasbourg, F-6700 Strasbourg, France; 4 Lorraine University Medical School, F-54500 Vandoeuvre-Les-Nancy, France

**Keywords:** Mobility, Exposure, Air pollution, Nitrogen dioxide, Birth outcomes

## Abstract

**Background:**

Prenatal exposure to outdoor air pollution has been shown to have health effects in many studies; low birth weight, preterm delivery, small for gestational age, and stillbirth are the most often cited. However, exposure of pregnant women is difficult to quantify, especially with regard to their mobility, which is rarely taken into account in epidemiological studies. This study aimed to assess the impact of mobility of pregnant women living in Paris, France, on their exposure estimates to nitrogen dioxide (NO_2_).

**Methods:**

A total of 486 pregnant women were recruited in 5 maternity hospitals in Paris between January and April 2016. A questionnaire was used to collect mothers’ characteristics (demography, education, etc.) and to assess their daily mobility during pregnancy (time spent at work, commuting time and mode used to move from residential to occupational places). Daily NO_2_ concentrations were estimated based on the combination of annual average concentrations modeled at the census block scale and daily concentrations measured from fixed monitoring stations. Different models were used to compare the exposure of pregnant women in residential and occupational places, also taking into account travel time and travel mode. The socioeconomic profile of the census blocks was characterized using a multi-component index.

**Results:**

During the first trimester of pregnancy, women living in the least deprived census blocks were exposed to higher concentrations of NO_2_ than those living in the most deprived ones. Occupational mobility had a small impact on exposure levels (average increase after taking account of mobility: + 0.52 μg/m^3^) which was not related to the socioeconomic profile of the women. The commuting mode made a greater difference (+ 1.46 μg/m^3^ on average), in particular among women living in the most deprived census blocks.

**Conclusions:**

Our study illustrates that air pollution exposure can be underestimated when ignoring occupational mobility and commuting mode of pregnant women. This effect might be differential according to the neighborhood deprivation profile.

**Electronic supplementary material:**

The online version of this article (10.1186/s12940-018-0416-8) contains supplementary material, which is available to authorized users.

## Background

Urban outdoor air pollution has many health effects, such as respiratory and cardiovascular diseases [1–4]. According to the World Health Organization, around 3 million premature deaths a year were linked to exposure to outdoor air pollution in 2012 [5]. Vulnerable groups including children, the elderly and pregnant women should be given special attention. Many studies and reviews suggest an impact of air pollutants on birth outcomes, particularly on low birth weight (LBW), preterm birth (PTB), small for gestational age (SGA) and stillbirth [2, 6–8]. Particulate matter (PM), particularly PM_2.5_, is of special concern, mostly for LBW, PTB, SGA and stillbirth [9–14] but nitrogen oxides (NOx) are also of concern [8, 15]. The impact of air pollution on fetal growth was studied in a prospective birth cohort in Los Angeles, California [16]. The authors showed that prenatal exposure to traffic-related pollution, estimated using air dispersion modeling for NOx, was negatively associated with fetal head size measured as biparietal diameter in late pregnancy. However, results differ across published studies, discrepancies that might be due to difficulties in quantifying exposure, to differences in exposure assessment methods, in time of measurement, and to collinearity between pollutants [2].

Several studies focused on the residential mobility of pregnant women. According to the review of Bell and Belanger (2012), the percentage of women who moved during pregnancy ranged from 9 to 32%, with a median of 20% among the 12 studies presenting this information in the United States (7 papers), the United Kingdom [2] and Australia, Canada and Norway [17]. However, daily mobility of pregnant women across the study area is also an important issue but this factor is rarely taken into account in epidemiological studies. Daily exposure to nitrogen dioxide (NO_2_) in Montreal, Canada, show significant differences between dynamic and static approaches to exposure assessment [18]. When considering their mobility across the city, the authors observed that most individuals had a higher daily exposure compared to the daily average concentration at their home location. In another study, Dhondt et al. (2012) found that the health impact of NO_2_ using an exposure metric that integrates time-activity information was on average 1.2% higher than when assuming that people stay at their home address [19].

In this context, our study aims to assess the impact of daily mobility on exposure to NO_2_ of pregnant women living in Paris, France, in particular through integrating the time spent at work locations and while commuting. For this purpose, NO_2_ concentrations modeled at a fine geographical scale (the census block) were used to compare exposure of pregnant women in the places of residence and of occupational activity. Also, commuting time and commuting mode used to move from the place of residence to the job location were two parameters introduced in the exposure models. Mobility among pregnant women was examined according to the SES profile defined at individual level and at residential census block in view to explore effect modification, i.e. whether the impact of mobility differed according to the socioeconomic profile of pregnant women.

## Methods

### Study settings and small area

The city of Paris has a population of about 2,250,000. The small-area level used was the IRIS (a French acronym for defining a spatial scale comparable to the census block). Designed by the French National Census Bureau (INSEE), the IRIS constitutes the smallest census unit area whose aggregate data, including socioeconomic information, can be used on a routine basis. The city of Paris is subdivided into 992 IRIS with a mean population of 2199 inhabitants and a mean area of 0.11 km^2^.

### Individual data

**Fig. 1:**
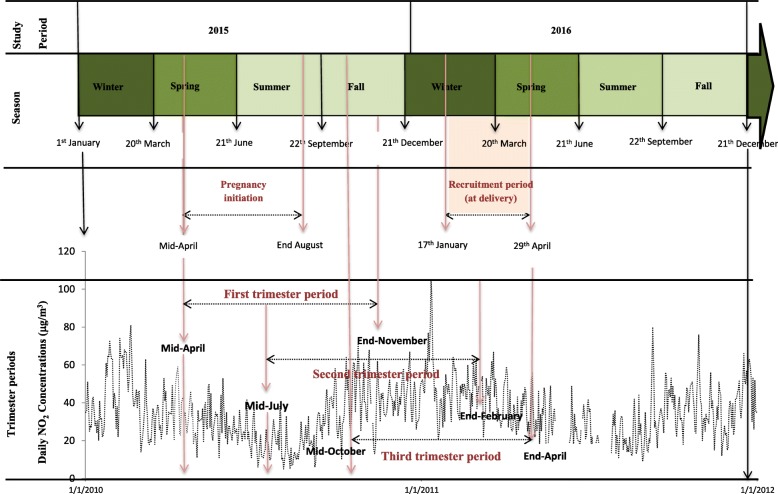
Calendar of women recruitment and associated pregnancy periods; As an illustration, time series of daily NO_2_ concentrations is obtained using data from the monitoring station located in the 6th arrondissement of Paris city

**Fig. 2:**
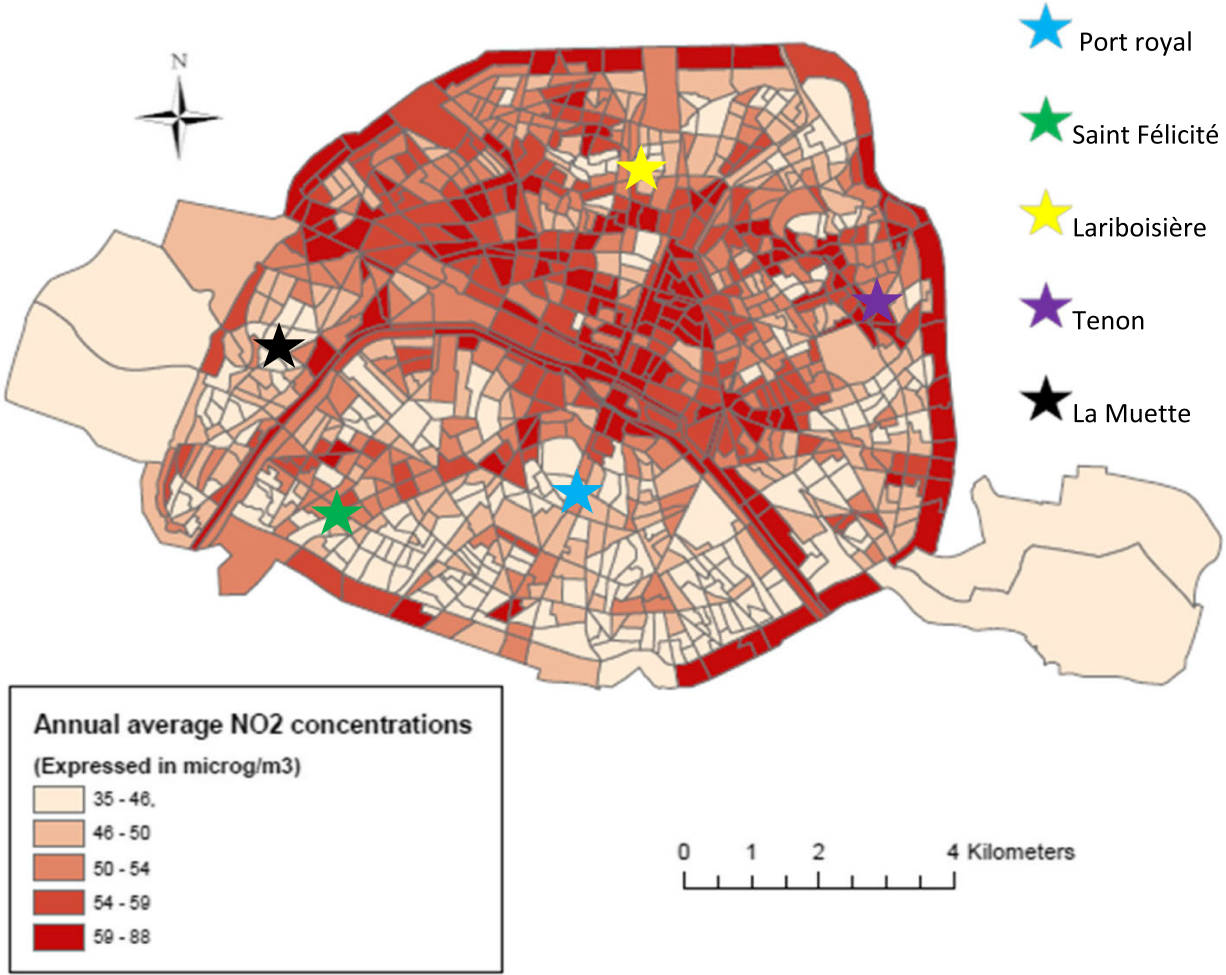
Spatial distribution of the annual average NO_2_ concentrations (in μg/m^3^) estimated by the air pollution model and categorized in quintile of its distribution (source: Airparif; period: 2010–2011) and the five maternity hospitals included in the study

Figure 1 describes the women recruitment calendar and the timing of pregnancies across the study period. The recruitment period spanned from 17th January to 29th April 2016 in 5 maternity hospitals of Paris: Port-Royal, Lariboisière, Tenon, Sainte-Félicité and La Muette (they are geolocated in Fig. 2). The sampling approach was pragmatic. In view to have a wide coverage of spatial and socioeconomic characteristics of women, the maternity hospitals were chosen, in collaboration with the Paris city department of mother and child health (PMI, Protection Maternelle et Infantile in French), according to five criteria: (i) they are scattered across the city of Paris (see Fig. 2) and (ii) sitting in areas with different socioeconomic profiles, based on our previous work on the link between exposure to environmental risk factors and socioeconomic deprivation across the city of Paris [20] (deprived, *n* = 2; more well-off, n = 2; intermediate, *n* = 1, the largest Paris maternity hospital, Port-Royal); (iii) their yearly number of deliveries is ‘large’ (i.e. > 1000; for instance, more than 5000 deliveries take place per year in Port-Royal); (iv) they were either public (*n* = 3) or private (n = 2); (v) finally, they accepted to host a trained investigator (RF) in charge of distributing, collecting and registering the two questionnaires during the study period: one questionnaire was used to describe the mothers –demographics, education etc. – the other one was used to assess in detail the mobility pattern of each study participant (where, how long, how).Participation of the maternity hospitals was planned as alternative weeks or days so as to spread inclusions across the whole study period for each maternity, except for one (Sainte-Félicité) which could only participate in winter and early spring, due to moving to another close location. All mothers coming to deliver during the days planned for their maternity were invited to inform the questionnaires (or answer the questionnaire-based interview with the investigator, should they prefer so; this mainly applied to women who were unfamiliar with questionnaires and/or had difficulties to read; in all cases, the investigator had been trained not to influence the answers) if they resided, within Paris, in one “arrondissement” adjacent to where the maternity was located; participation was entirely on a voluntary basis. Women were informed they could decline participation to the study. Any information which could be used to identify participants (home or work address) was erased after census block/IRIS coding.

### Contextual data

#### Air pollution

In our study, occupational and residential addressees of pregnant women were geocoded at the centroid of the census block(s) of their place of residence or of occupational activity. Therefore, to assess cumulative exposure of pregnant women taking into consideration occupational mobility, we retrospectively estimated daily concentrations of NO_2_ at the census block level.

Daily concentrations of the study pollutant in each census block were estimated based on the combination of the annual average concentrations modeled at the census block level with the daily variations of its index monitor (more details are given in Deguen et al. 2015 and Kihal et al. 2016) [20, 21]. To do so, two types of air pollution data available for the years 2010 and 2011 were used: (i) data from monitoring stations and (ii) data modeled by the air quality monitoring networks of the Ile-de-France Region (AirParif). In the present study, indoor concentrations were not available. Several studies have shown that outdoor and indoor concentrations of NO_2_ are of the same order of magnitude and the median ratios indoor/outdoor are close to 1 [22, 23].

(i) Daily nitrogen dioxide (NO_2_) concentrations were available from fixed monitoring stations (both from background and traffic stations) located within the city of Paris.

(ii) Annual average ambient concentrations of NO_2_ were modeled for each census block. AirParif used a deterministic model named ESMERALDA (www.esmeralda-web.fr) which integrates various input parameters including linear (main roads), surface (diffuse road sources, residential and tertiary emissions) and industrial point sources, and meteorological data [24]. More than 200 point sources were selected from the regional emission inventory. Emissions for road traffic were estimated combining the regional traffic network and the COPERT III European database over the period 2002–2006, and COPERT IV over the period 2007–2012. Concerning meteorological data, the Mesoscale Meteorological model (http://www2.mmm.ucar.edu/mm5/) was used. The NO_2_ background concentrations were determined by combining monitored NO_2_ concentrations from monitoring stations and those modeled at a regional scale from the ESMERALDA. The NO_2_ road traffic concentrations estimated from the STREET software model using more than 36,500 sections of roads were added to NO_2_ background concentrations.

We also used the standard deviation of the annual average ambient concentrations of NO_2_ (based on values estimated at a 25 m spatial resolution) to analyze the variability of NO_2_ within census blocks.

To estimate the daily concentration of NO_2_ at the census block level (residential and occupational), two additional steps were required: *1*) to assess the spatial area representative of the air quality monitoring stations in the study area. *2*) to reconstitute the daily concentrations of air pollutant at the census block level.

##### Step 1: To assess the spatial area representative of the air quality monitoring stations in the study area

Using daily NO_2_ concentrations measured by 7 fixed monitoring stations (including background stations and traffic stations) located within the city of Paris and available over the 2010–2011 period, we assigned to each census block the air quality monitoring stations which provided the best estimates of daily concentration variability. To do so, a hierarchical agglomerative clustering (HAC) was applied which allowed grouping census blocks and stations into clusters (groups) based on similarities within a cluster and dissimilarities between clusters. The HAC analysis detected seven clusters of census blocks and their associated monitoring stations. If more than one monitoring station defined one cluster, the best representative air quality monitoring station for the census block is selected according to its spatial proximity. Therefore, each census block was assigned to the monitoring station (named the “index” monitor) best representing the NO_2_ variation within the census block.

##### Step 2: To reconstitute daily variations of air pollutant concentrations at the census block level

Using the Pregnancy Air Exposure R package [25] we combined the annual average concentrations of NO_2_ modeled at the census block scale with the relative daily variations to the annual average of its index monitor (as the index monitor is assumed to be representative of the daily variations of NO_2_ within the census block). For instance, if, for a given day, the index monitor measured that daily concentrations of NO_2_ were 12% lower than its annual average, the daily concentration in this census block is set 12% lower than its annual average concentration. However, when monitoring stations series contain missing values, imputation function must be applied before using the Pregnancy Air Exposure R package. The imputation method takes account for temporal dimensions, correlations between measurements in different monitoring stations, and the log-normality of the data [26].

#### Socioeconomic deprivation index

A deprivation index was used to capture different socioeconomic dimensions by combining variables (family structure, household type, immigration status, employment, income, education and housing) collected by the National Institute of Statistics and Economic (INSEE). Several successive principal component analyses were run to combine the socioeconomic variables in a composite index. Briefly, a Principal Component Analysis (PCA) was used to select 15 variables among 41 initial socioeconomic and demographic variables provided by the 2012 national census at census block level. The 15 variables were those the most correlated with the first principal component. A final PCA was used to calculate the socioeconomic deprivation index. Developed by Lalloué et al. in 2013, this index proved its validity to analyze environmental and health inequalities in France and more specifically in Paris [27]. This deprivation index was categorized into 5 classes of census blocks according to the quintile of its distribution.

### Statistical analysis

NO_2_ exposure during the two first trimesters of pregnancy was estimated in four different ways (Eqs. 1 to 4). For each birth, a proxy of individual exposure by trimester of pregnancy was derived using the date of birth, the gestational age, and the census block of residence. Daily concentrations of NO_2_ at residential and/or occupational activity census blocks were averaged over each trimester of pregnancy. First and second trimesters were defined as the weeks between 1 and 13 of pregnancy and between 14 and 26, respectively; the third trimester was not included in our study due to a limited number of women working during this last pregnancy period.

In Eq. 1, we postulate that pregnant women stay the entire day in their residential census block while in Eq. 2, we take into account that, on average, pregnant women spend 31% of their time in the census block where they work according to the Eurostat figures (http://ec.europa.eu/eurostat/fr/data/database). In Eq. 3, we introduce the fact that pregnancy women, as a majority of workers in France, work 5 days per week, and we make the assumption that during the 2 days off, they stay in their residential census block. Finally, time spent to commute from residential to occupation census blocks and travel mode was considered in Eq. 4. Doing so, we postulate that exposure during transit depends on commuting mode but does not vary across census blocks nor over time. More specifically the different indicators were constructed as follows.

#### Model 1 indicator

The first indicator of exposure, defining Eq. 1, takes only into account the place of residence of the pregnant women ignoring their daily mobility for occupational reasons. Exposure to NO_2_ of a pregnant woman during a given pregnancy trimester is then estimated by the following simple equation:

1 $$ {E}_{ik}={C}_{ijk} $$

- *Where C corresponds to the average of daily NO*_*2*_ *concentrations estimated in the residential census block j of pregnant woman i during trimester k (k={1;2})*

#### Model 2 indicator

The second indicator of exposure, defining Eq. 2, also takes into account the occupational census block knowing that, people on employment spend on average 31% of their time within the occupational census block. NO_2_ exposure of a pregnant woman during a given pregnancy trimester is then estimated by the following equation:

2 $$ {E}_{ik}=0.69\ast {C}_{ijk}+0.31\ast {C}_{iuk} $$

- *Where C corresponds to the average of daily NO*_*2*_ *concentrations estimated in residential j and in occupational u census blocks of pregnant woman i during the trimester k (k={1;2})*

#### Model 3 indicator

In Eq. 3, the two days off per week are considered, based on Eq. 2. NO_2_ exposure of a pregnant woman during a given pregnancy trimester is then estimated by the following equation:

3 $$ {E}_{ik}=\left\{0.69\ast {C}_{ijk}+0.31\ast {C}_{iuk}\right\}\ast \frac{5}{7}+\left\{{C}_{ijk}\right\}\ast \frac{2}{7} $$

- *Where C corresponds to the average of daily NO*_*2*_ *concentrations estimated in residential j and in occupational census block u of pregnant woman i during trimester k (k={1;2}) and,*
- *Weights [5/7] and [2/7] stand for the 5 working days and 2 days off of a given week, respectively.*

#### Model 4 indicator

Finally, in Eq. 4, commuting time and mode used to move from residential to occupational census blocks are two additional parameters introduced in Eq. 3. According to a study on city dwellers exposed to air pollutants in the Paris urban area, six distinct travel modes were considered, with associated median concentrations of NO_2_: metro (54 μg/m^3^), car (130 μg/m^3^), bus (140 μg/m^3^), bicycle (71 μg/m^3^), tramway (61 μg/m^3^) and by foot (56 μg/m^3^) [28]. We postulated that exposure during transit does not vary across the census blocks; the same level of exposure was assigned to women who use the same travel mode irrespective of the residential or occupational census block. The resulting NO_2_ exposure of a pregnant woman during a given pregnancy trimester is then estimated by the following equation:

4 $$ {E}_{ik}=\left\{\left(0.69-{t}_i\right)\ast {C}_{ijk}+0.31\ast {C}_{iuk}+{t}_i\ast {C}_{Ti}\right\}\ast \frac{5}{7}+\left\{{C}_{ijk}\right\}\ast \frac{2}{7} $$

- *Where C corresponds to the average of daily NO*_*2*_ *concentrations estimated in residential j and in occupational census block u of pregnant woman i during trimester k (k={1;2}),*
- *weights [5/7] and [2/7] stand for the 5 working days and 2 days off of a given week, respectively,*
- *t*_*i*_ *represents the proportion of the daily time spent to commute to the occupational census block by pregnant woman i, and,*
- ${C}_{T_i}$ *, the NO*_*2*_ *concentrations associated with the travel mode T of the pregnant woman i.*

Statistical analyses were realized with the Stata software (descriptive statistics and paired-difference tests) and the map representations with ArcGIS software. The statistical significance level was set to α = 5%. Differences between NO_2_ exposures according to models previously described were investigated by trimester of pregnancy and by deprivation category, separately.

## Results

### Study population description

In total, 504 women who gave birth at one of the participating hospitals or private clinics during the study period were eligible; among them, 486 accepted to be interviewed; the majority of the non-respondents were homeless women with a very deprived socioeconomic profile. Participating women were aged between 17 and 46 years old (Mean = 28.9 years; Standard deviation = 5.3 years). Main descriptive statistics are summarized in Table 1. About 40% of the study population has a high level of education (higher than the French baccalauréat, termination of secondary school). A majority of the women worked during the first trimester of pregnancy (80%) whereas less than a quarter did so during the third one. The majority of women traveled by metro to move from their residential to their occupational census block; the daily commuting time was equal to 45 min on average (standard deviation = 27 min); it decreases significantly (p-trend = 0.03) from women with the lowest level of education (average time = 51.5 min, SD = 24.1 min.) to those with the highest (average = 44.8 min, SD = 27.2 min.). While the residential census blocks of participation women cover 34.8% of the 992 census blocks of Paris, this percentage falls to 28.5% and 23.1% when considering the occupational census blocks during the first and the second trimester of pregnancy, respectively.

**Table 1:** Descriptive statistics of the study population

Characteristics (*N* = 486)	N	%
Hospital/clinic		
Tenon	115	23.7
Lariboisière	105	21.6
Port-Royal	173	35.6
Sainte-Félicité	80	16.5
La Muette	13	2.7
Education		
Low (<Baccalaureat)	132	27.2
Middle (= Baccalaureat level)	159	32.7
High (> Baccalaureat)	195	40.1
Census block socioeconomic deprivation class		
(most privileged) 1	96	19.7
2	96	19.7
3	92	18.9
4	111	22.8
(most deprived) 5	75	15.4
*Missing value* ^a^	*16*	*3.3*
Occupational activity		
1st trimester (T1)	398	81.9
2^de^ trimester (T2)	300	61.7
3rd trimester (T3)	112	23.0
Commuting modes		
By foot	49	10.1
Bicycles / motorcycles	9	1.9
Car	10	2.1
Metro	320	65.9
Bus	51	10.5
Tramways	2	0.4
*Missing value*^b^	*45*	*9.3*
IRIS of residence (*N* = 992 IRIS)	345^c^	34.8^c^
IRIS of occupation (N = 992 IRIS)^d^		
1st trimester (T1)	283	28.5
2^de^ trimester (T2)	229	23.1
3rd trimester (T3)	91	9.2

^a^It was not possible to geocode sixteen women due to incomplete postal address

^b^Missing/no information regarding commuting mode

^c^The 486 women were distributed across 345 census blocks, representing 34.8% of the total number of census blocks in Paris (345/992)

^d^Nine women had the same residential and occupational census blocks (IRIS)

### Spatial distribution of the study population

**Fig. 3:**
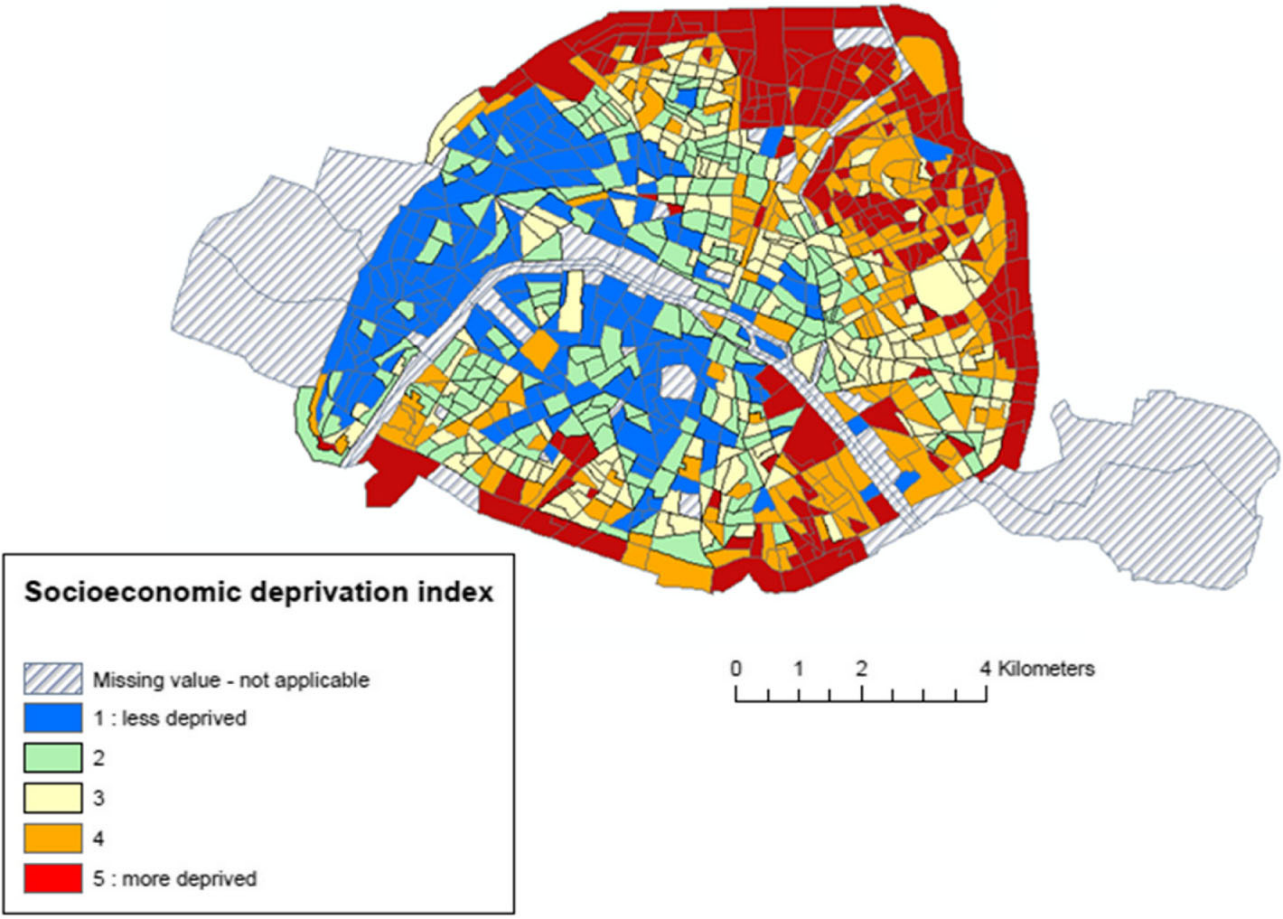
Spatial distribution of the deprivation index categorized in quintiles (source: INSEE 2012)

**Fig. 4:**
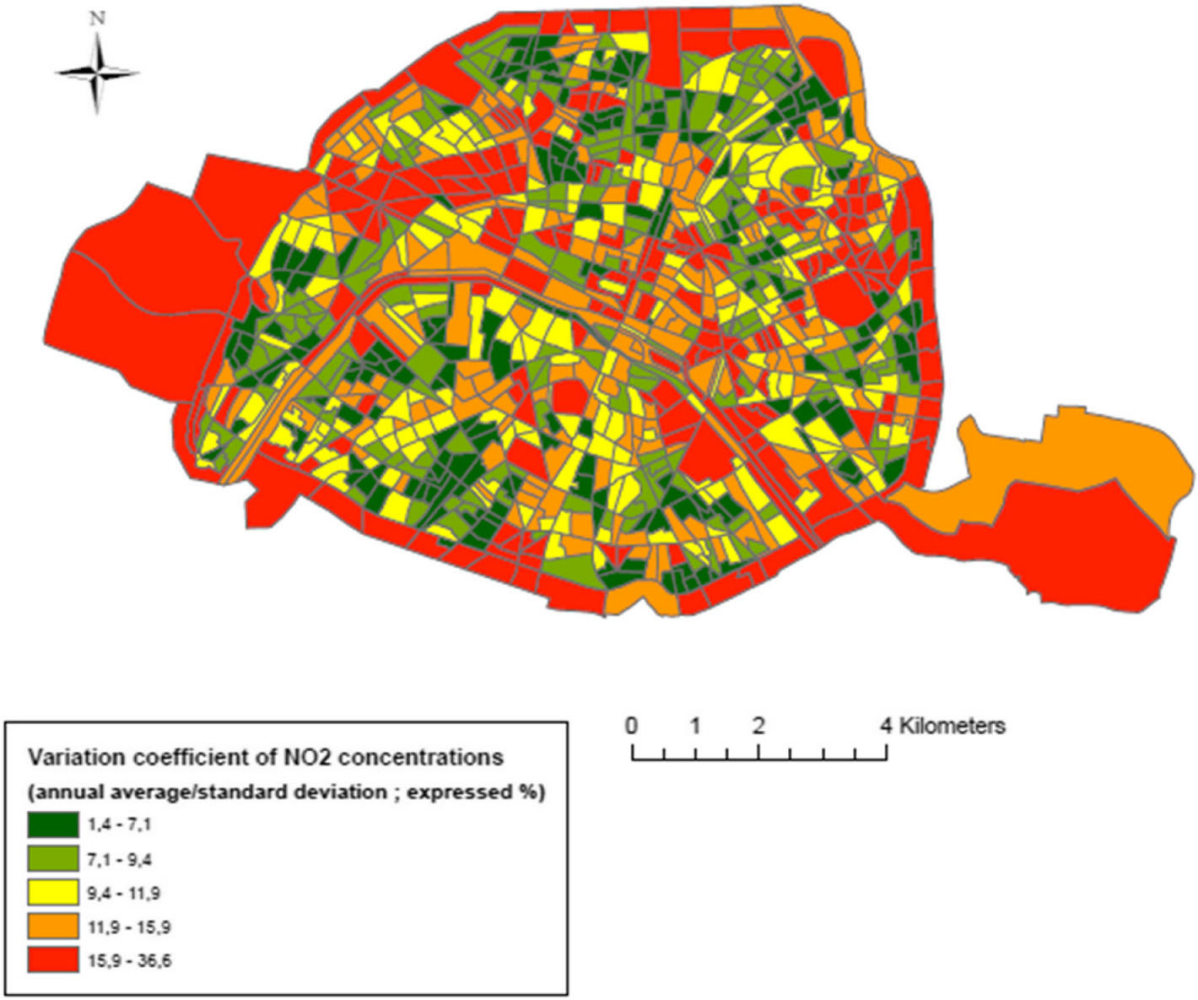
Spatial distribution of the variation coefficient of NO_2_ concentrations (Standard deviation/Annual average; expressed in percent) categorized in quintile of its distribution (source: Airparif; period: 2010–2011)

The spatial distribution of the NO_2_ concentrations (Fig. 2) highlight a gradient from the South of the Seine River, with lower levels (the majority of the census blocks exhibit values of less than 50 μg/m^3^), to the North, with higher levels (the majority of the census blocks, in dark colors, show values greater than 50 μg/m^3^). NO_2_ concentrations vary also according to season: greater concentrations are experienced in autumn and winter compared to spring and summer (Fig. 1). The spatial distribution of the socioeconomic index (Fig. 3) reveals a clear gradient from South-West, that host the less deprived areas, to North-East, the most deprived ones (census blocks colored in red in Fig. 3). The variation coefficients estimated at the census blocks level (standard deviation/ annual average of NO_2_ concentrations) vary between 1.4% (minimum) and 36.6% (maximum), with a median value of 10.5%; Fig. 4 exhibits its spatial variability: The variation within census blocks is higher in those close to the ring highway around Paris and in the center of the city near the high traffic avenues. Analyses of the variation coefficient per group of socioeconomic deprivation show no significant trend (p-trend = 0.22).

**Fig. 5:**
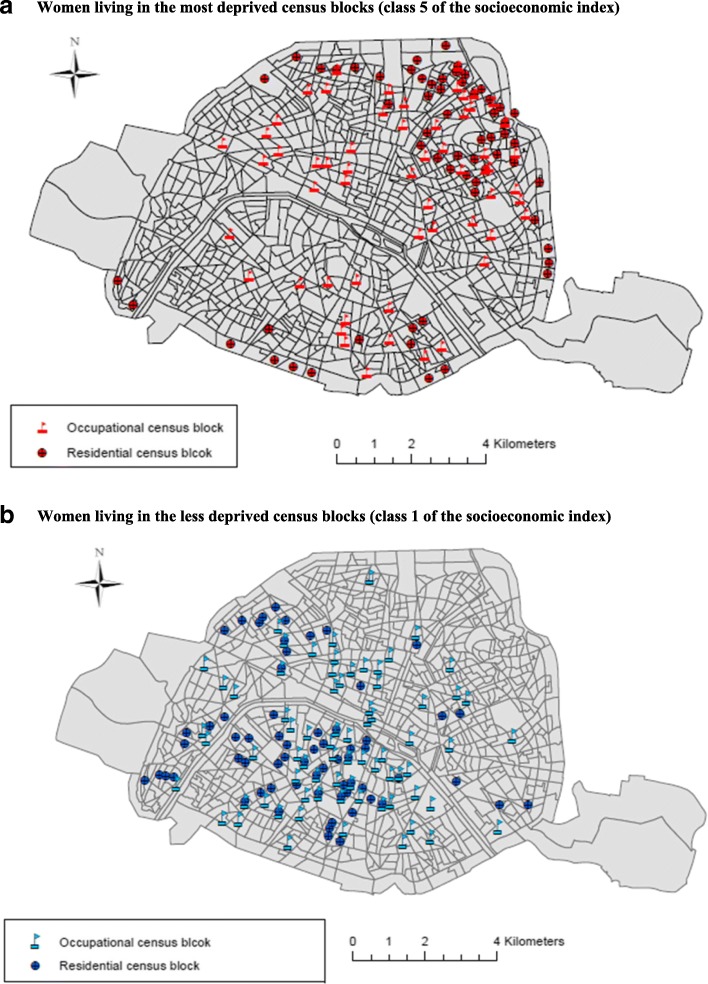
Spatial distribution of the residential and occupational census blocks (dark and light color respectively) of the study population living in the most deprived (map *a*- colored in red) and the less deprived (map *b*- colored in blue) census blocks

Figure 5a and b exhibit the spatial distribution of the residential and occupational census blocks of the study population living respectively in the most deprived and the most privileged areas. They show that women living in the most deprived census blocks (mainly North and East areas) work in census blocks located more frequently in the center of Paris where concentrations of NO_2_ are the highest. On the other hand, women living in the most privileged census blocks work in their great majority in the same census block or close to it.

Table 2 further describes women who have occupational activities during the first, second and third trimesters, according to the socioeconomic profile of their place of residence. While always more than 80% of pregnant women work during the first trimester of their pregnancy, this proportion reduces to about 60–70% during the second trimester and flattens to less than 30% during the last trimester, with a trend towards a higher level of occupational activity among women living in less deprived blocks.

**Table 2:** Occupational activity of women per socioeconomic deprivation class of their residential census block, according to the trimester of pregnancy

Deprivation classes	C1 (less deprived)	C2	C3	C4	C5 (more deprived)
N (%)	96 (100%)	96 (100%)	92 (100%)	111 (100%)	75 (100%)
1st trimester	86 (90%)	79 (82.3%)	80 (87%)	91 (82%)	62 (82.7%)
2nd trimester	65 (67.7%)	60 (62.5%)	63 (65.6%)	68 (61.3%)	44 (58.7%)
3th trimester	26 (27%)	23 (24%)	27 (28%)	20 (18%)	16 (21.3%)

### Patterns of NO_2_ exposure during pregnancy

NO_2_ concentrations at the residential and occupational census blocks show comparable levels (Table 3). The values increase with the trimester of pregnancy, in relation with the main emission sources that may vary depending on the season, and span between a minimum and a maximum about 25 and 90 μg/m^3^, respectively, with a median value about 45 μg/m^3^.

**Table 3:** Descriptive statistics of NO_2_ concentrations (in μg/m^3^) estimated at census blocks of residence and occupation of the study women, separately, by trimester of pregnancy

	Trimesters exposure	Minimum	5th percentile	Median	95th percentile	Maximum	N
Census blocks of residence	T1	25	28	42	58	79	470
	T2	34	40	51	66	85	470
	T3	42	48	57	67	89	470
Census blocks of occupation	T1	25	29	44	59	65	398
	T2	35	42	53	70	78	300
	T3	44	47	58	71	84	112

**Fig. 6:**
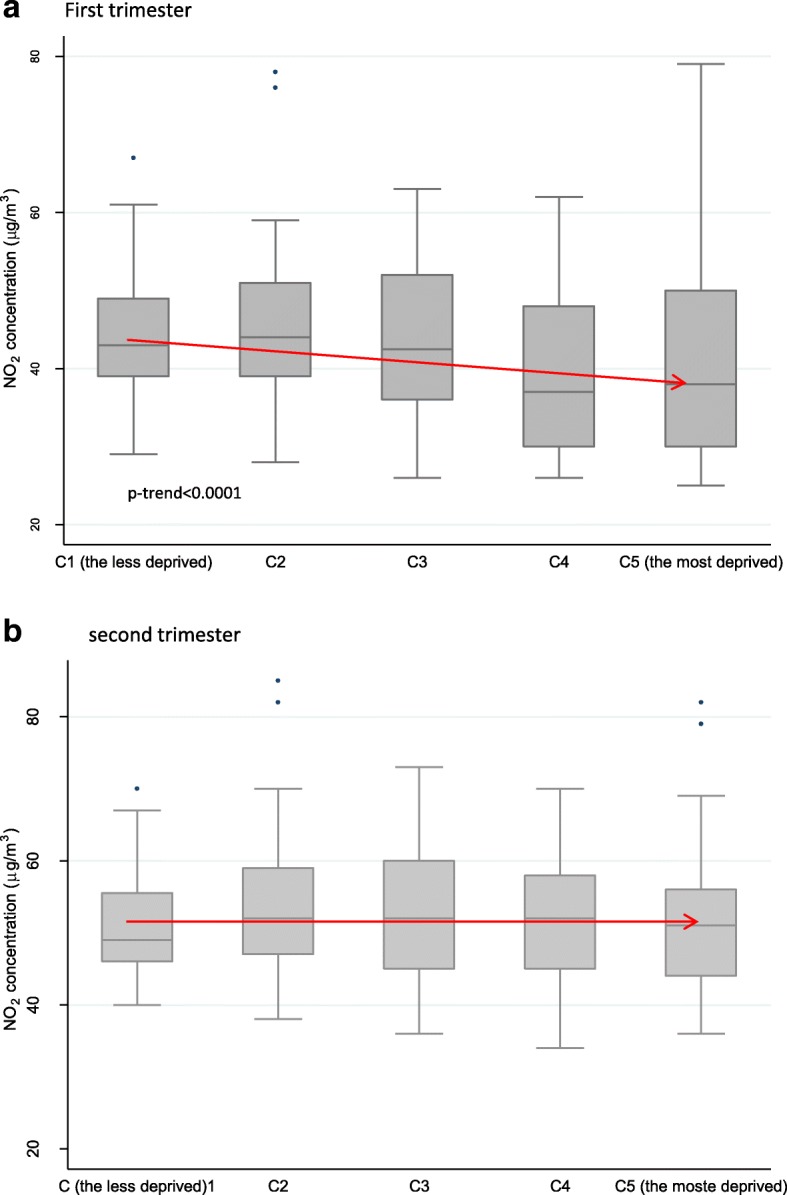
Distribution of NO_2_ concentrations (in μg/m^3^) estimated at the residential census blocks, by class of deprivation, during a- the first trimester of pregnancy and b- the second trimester of pregnancy

Exposure levels exhibit socioeconomic patterns. During the first trimester, women living in the privileged census blocks are exposed to higher concentrations of NO_2_ at their place of residence than those living in the most deprived ones, with a significant trend across the census blocks socioeconomic score (p-trend< 0.0001) (Fig. 6a). There are also differences in the variability of the NO_2_ concentrations according of the socioeconomic profile of the census blocks: although weaker on average, the most deprived ones exhibit greater ranges, with values spanning from 50 μg/m^3^ to 79 μg/m^3^ (the maximum value) in the last quartile of the distribution (Fig. 6a).

Relations between NO_2_ exposure from Eq. 1 and the three other models are described by Additional file 1: Figure S1. The correlation coefficient quantifies how close NO_2_ exposure estimates between two models are. In other words, it is a measure of the misclassification of exposure estimate when ignoring daily mobility (Eqs. 2 and 3) and the travel mode (Eq. 4): the closer to 1 the correlation coefficient, the smaller the degree of misclassification of NO_2_ exposure. There is very little difference between exposure estimates of Eqs. 2, 3 or 4, compared to Eq. 1. We will however see further that model 4 sharpens the contrasts between census blocks, according to their socioeconomic characteristics.

**Fig. 7:**
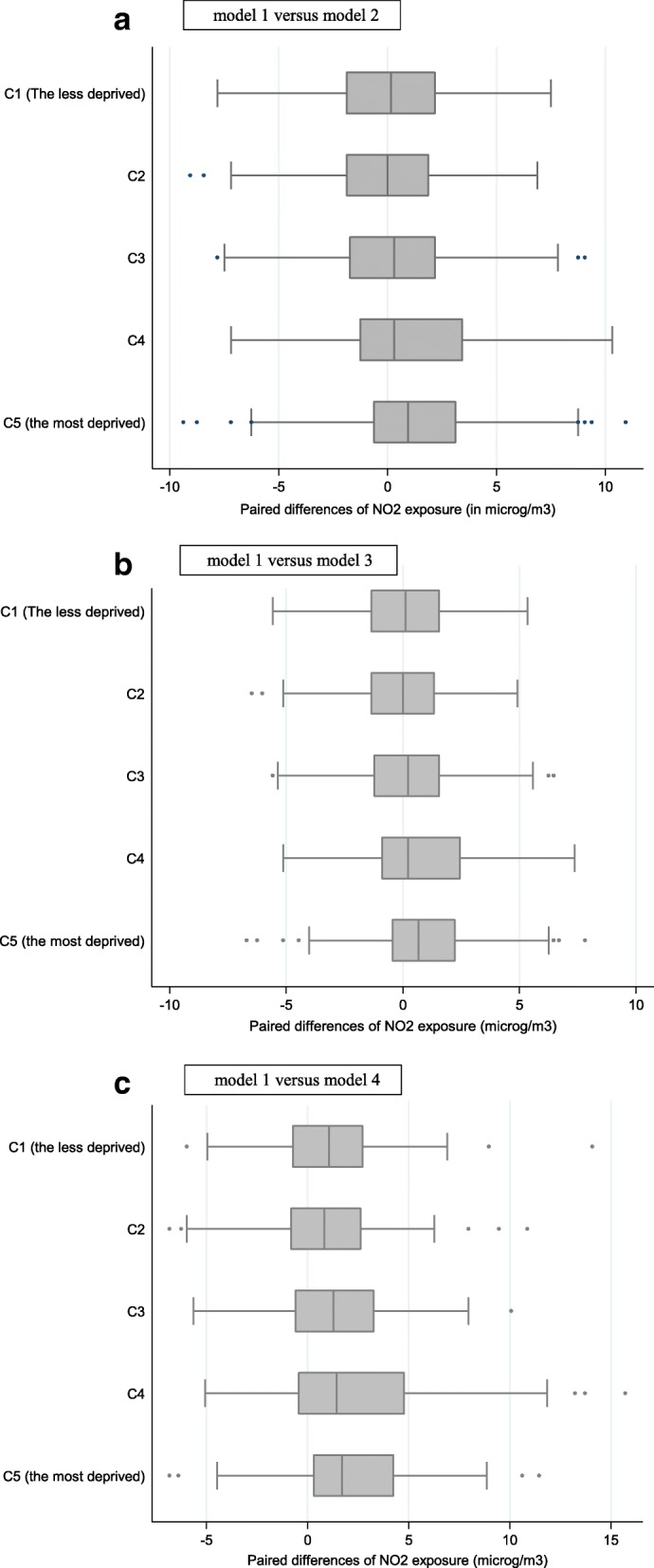
Paired differences of NO_2_ exposure (in μg/m3) estimated during the first trimester of pregnancy between models 1 and 2(**a**), models 1 and 3(**b**), and models 1 and 4(**c**), by class of deprivation

Occupational mobility has an impact on exposure levels: during the first trimester of pregnancy, the paired differences of NO_2_ estimates were all significantly greater than 0 when contrasting Eq. 1 with respectively Eqs. 2, 3 and 4. These differences are small, however, with average differences = 0.52 μg/m^3^ (SD = 3.7 μg/m^3^); 0.37 μg/m^3^ (SD = 2.7 μg/m^3^); and 1.46 μg/m^3^ (SD = 3.3 μg/m^3^), respectively. But this impact of occupational mobility varies across the exposure range: among women whose exposure estimates is the least altered when considering occupational mobility (the lowest quartile of the distribution of paired differences between Eqs. 1 and 4), it tends to reduce exposure estimates, while among women whose exposure estimates is the most altered when considering occupational mobility (the highest quartile of the distribution of paired differences), it tends to increase exposure estimates. This is not so much associated with the socio-economic profile of the census block than with the commuting time and mode: the paired differences between NO_2_ exposure estimates from Eqs. 1 and 2, and respectively models 1 and 3, are only mildly linked to the census block socioeconomic profile (p-trend = 0.13 and 0.14, respectively, Fig. 7a and b). On the other hand, Fig. 7c suggests that considering exposure during commuting makes a difference, with women who live in the most deprived census blocks (class 5) showing the highest paired exposure differences between Eqs. 1 and 4 that (accounts for occupational mobility plus travel time and travel mode), followed respectively by deprivation classes 4 and 3 (p-trend = 0.04).

**Fig. 8:**
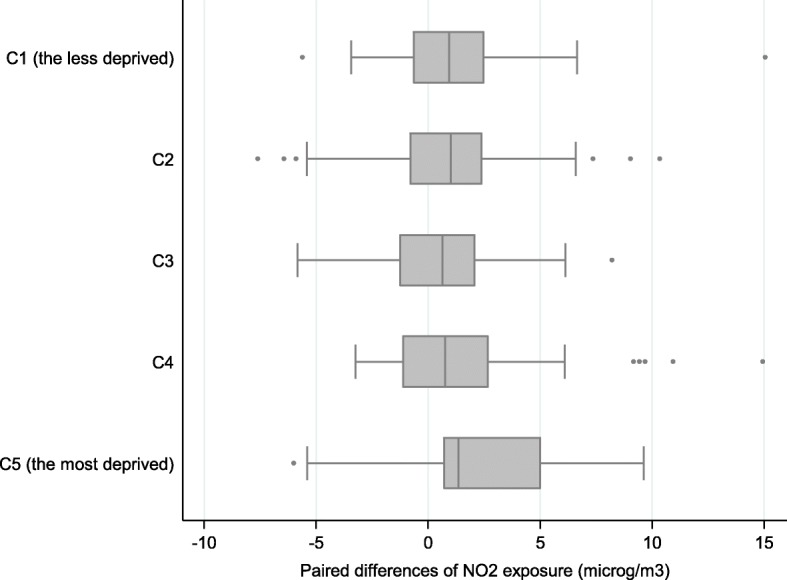
Paired differences of NO_2_ exposure estimated during the second trimester of pregnancy (in μg/m3) between models 1 and 4 by class of deprivation

This finding could reflect the commuting time that is significantly higher (p-trend = 0.02) for women living in the most deprived census blocks compared to those living in the most privileged census blocks, as stated above. No significant differences were revealed during the second trimester of pregnancy, when occupational mobility is less frequent (Fig. 8).

## Discussion

Our study on pregnant women in Paris reveals that daily mobility associated with occupation, in particular through integrating the time spent at work locations and while commuting, may increase the exposure estimates to NO_2_, an indicator of air pollution. However small in absolute terms (around 0.5 to 1.5 μg/m^3^ on average), the impact of daily mobility on exposure estimates are not benign in relative terms, considering that these differences may amount respectively to 31 and 94% of the NO_2_ concentration contrasts (during the first trimester) between the extreme quintiles of the distribution across census blocks. In addition, we found that the impact of daily mobility is more important when focusing on women who live in the most deprived census blocks.

Several studies have investigated how population mobility could modify the estimates of air pollution exposure. As a general rule, studies on outdoor air exposures during pregnancy use air pollution data estimated at residential locations. In a recent study, Shekarrizfard et al. observed that taking into account individual mobility across the city increases the estimates of daily NO_2_ exposure compared to the average NO_2_ concentration at the home location [18]. Setton et al., in 2008 [29], confirmed previous findings of the study by Marshall et al., in 2006 [30] demonstrating that although the time spent at home contributes most to exposure differences among census tracts, time spent at work locations explain the within-census tract variability in exposures. These subtle changes may have consequences on effects measures in epidemiological studies. Dhondt et al. (2012) found an impact of NO_2_ on respiratory mortality on average 1.2% higher using an exposure that integrates time-activity information than when assuming that people stay at their home address [19]. In another study Setton et al. (2011) posit that exposure assessment that would only take into account air pollutants concentrations at home locations is a static approach which could lead to a bias in health assessment by ignoring individual travel patterns [31]. They found that ignoring daily mobility contributes to negative bias in relative risk estimates; comparing two study areas, they also revealed that this negative bias increases when pollutant concentrations are spatially heterogeneous compared to a more homogeneous area. Using individual data routinely collected via participants’ mobile phones, an innovative study [32] highlighted the importance of considering daily mobility in estimating the exposure to air pollution: the mean increase of NO_2_ exposure was equal to 4.3 and 0.4% during week days and weekend days, respectively, when individual daily mobility was accounted for, which resulted in an underestimation of the health effects of NO_2_. Finally, a recent study [33] stands that future research has to consider spatiotemporal variability of environmental risk factors and daily individual mobility in order to avoid misleading results in exposure assessment.

While several recent studies document the importance of considering daily mobility in individual exposure estimate, our study is the first, to our knowledge, which shows a differential impact of daily mobility according to socioeconomic characteristics. As a consequence, ignoring daily mobility, and in particular commuting modes, might lead to differentially misclassify exposure of the population subgroups according to social characteristics and might in turn bias the relative risk estimates in epidemiological studies. Daily mobility is of special relevance for exposure estimation in Paris where the spatial variability of NO_2_ concentrations across census blocks is great and is highly associated with the socioeconomic profile of the residential census blocks: on average, the NO_2_ concentrations at the Paris residential census blocks are the lowest in the most deprived census blocks, as stated above, a pattern that is not common in the country and that is due to the urban socioeconomic make-up of the city and its metropolitan area over time [34].

We propose the following hypothesis about this finding. If including occupational mobility in exposure estimates significantly reduces these estimates, it means that exposure during transit is lower, on average, than exposure at the residential census block (Eq. 1). This is more likely to occur among women who live in more polluted areas (who, in the context of Paris, is more frequently the case of more well-of census blocks) and who use commuting modes with relatively low pollution levels (eg walking, tram); this is consistent with the observation that women from more well-of census blocks spend less time to commute to their work location compared to women living in the most deprived census blocks, with a significant trend according to the deprivation index (p-trend = 0.02); Figs. 5 and 6 show that working women from more privileged area tend to have work places closer to where they live than working women from disadvantaged areas. Now, longer transit time also means more exposed commuting modes, as car or bus.

Exposure during commuting not only depends upon commuting modes, but also varies spatially, in particular (but not only) according to the travel routes and the time spent. Unfortunately, modeling travel routes was not possible in our study because this information was not collected by the questionnaires and because a typical route by bus or car in Paris is very non-linear. Regarding travel modes in Paris, expressed in terms of time spent while commuting for occupational reasons, the study pregnant women declare tram as the main mode (36% of the time while commuting, as median value), followed by metro and bus (16% in both cases for occupational reasons during week days), and biking (12%), then car (12%), with walking coming last (8%).

Several features of our studies should be considered to interpret correctly its findings. Firstly, the period over which the pregnant women were recruited has an impact on the results. In our study, for practical reasons linked with when it could be undertaken, the first pregnancy trimester occurred during the period from mid-April to end-November, i.e. when the NO_2_ concentrations are lowest in Paris (Fig. 1). The main emission sources of nitrogen oxides are road traffic (56%) and residential sector (18%), with a more marginal contribution of energy production (5%) and manufacturing industry (5%) [35]. During summer, NO_2_ concentrations are lower, due to the slowdown of activities in the city and in particular the decrease of road traffic associated with the holiday period, but also in link with the chemistry of ozone formation. Figure 2 shows a significant difference on NO_2_ concentrations during the first trimester of pregnancy according to the deprivation index with categories C1, C2 and C3 exhibiting greater values than C4 and C5. By contrast, during the second trimester, no difference is observed by census block score of deprivation (Fig. 3). Our results would probably have been different had the recruitment period been shifted in time. In addition, the collection of individual data was based on a pragmatic sampling approach which could introduce a selection bias in the study population. However, a comparison of the level of education (one important parameter in our study) with those of the Parisian population shows a strong similarity. The Ile de France region is one of the French regions where the proportion of the population with a high level of education is the greatest: in 2012, about 35% of the population living in Ile de France region had a university level of education (https://www.insee.fr/fr/statistiques/1288219). This is in accord with the high proportion of women with a high level of education, equal to 40%, in our study population.

Secondly, our finding is related to the specificity of the study area and its spatial organization. More precisely, the spatial distribution of the employment opportunities in a given area can influence the population mobility patterns by, for instance, increasing or decreasing travel time, and by modifying the transport mode used to commute to work location. In Paris, there is a high number of job opportunities in the central arrondissements [36], where the concentrations of NO_2_ are the highest. In our study, we found that the time spent commuting was higher for pregnant women who live in the most deprived census blocks located in the border of Paris, far from their workplace; this may explain why the combination of time spent and transport mode increases their personal NO_2_ exposure estimates. Exposure of commuters in the different modes of transport strongly depends on the time spent but also on the configuration and the types of commuting mode developed in each city or metropolitan area. A systematic review of 39 studies comparing exposure to air pollution according to different modes of transport has shown that commuters using motorized transports had highest levels of exposure compared to cyclists and pedestrians [37]. NO_2_ concentrations measured in the different mode of transport in Paris and used in this work show similar results [28]. A recent study of air pollution by mode of transportation during rush hours conducted in Montreal in 2016 gives NO_2_ concentrations of the same order of magnitude as those measured in Paris in 2007 and 2008 during the winter period [38]. In order to assess whether our findings were robust, we compared them with exposure estimates derived from NO_2_ concentrations measured in similar commuting modes in the Montréal city. Additional file 1 Figure S2 summarizes the distribution of NO_2_ exposure estimates when considering the travel (Eq. 4), once the NO_2_ concentrations measured in the different commuting modes in Montréal (respectively at the 5th, median and 95th values of the distribution) were substituted to those applied to the Paris pregnant women population. The result show similar patterns when comparing our initial results with those obtained with the median values of the Canadian study.

The impact of daily mobility on exposure has been recognized to increase with the level of NO_2_ spatial heterogeneity [33], a finding which is directly related to the smallness of the spatial unit. Our analysis is conducted at a small spatial scale, the census blocks with a mean population of 2199 inhabitants and a mean area of 0.11 km^2^, allowing to highlight large spatiotemporal variations of NO_2_ concentrations, a favorable situation to reveal the impact of daily mobility. However, due to the specificity of the Paris urban setting, it is not possible to draw a general statement from our findings because the literature shows a clear evidence of city-specific spatial and temporal environmental inequalities that relate to the historical socioeconomic make-up of the cities [34]. More precisely, the shape of the relationship between NO_2_ concentrations and socioeconomic characteristics measured at the census block level may be different according to the study area. For example, to remain in the French setting, in the Lille metropolitan area, the situation is at odds with that of Paris while in the Lyon metropolitan area there is no evidence of a gradient (non-linear relationship): the midlevel deprivation census blocks are the most exposed to traffic-related air pollution, supporting previous observations from the area of Strasbourg metropolitan area [39]. Similar discrepancies are observed elsewhere in Europe. While in Rome, Forastière et al. in 2007 [40] revealed a pattern close to the one found in Paris, where the less socioeconomically deprived census blocks experience higher levels of NO_2_, a reverse situation was described in Oslo by Naess and colleagues regarding exposure to fine particles [41]. Hence, daily mobility may differently impact personal exposures according to the urban setting, and the issue is to be assessed specifically in each study area.

Another limitation concerns the impact of indoor air pollution on our results. An infiltration factor of 0.66 was estimated for NO_2_ in different types of indoor environments in Sweden [22]. The results of this study also estimated a median indoor/outdoor ratio of 0.92, indicating that indoor air concentrations could be compensated by the different indoor sources; a similar ratio is also cited by WHO [23]. To apply this infiltration factor to the ambient NO_2_ concentrations in our data would have no impact on the results of Eqs. 2 and 3 because this correction would equally reduce NO_2_ concentration sat home and in the workplace. By contrast, Eq. 4 would be impacted: the relative contribution of indoor environments (home and work) to the overall exposure estimate would be lower, which would tend to increase the relative part of exposure while commuting. This reinforces our finding that exposure while commuting (in terms of duration and transport mode) has a more critical play in the differential impact of mobility according to the socio-economical profile of subjects (census blocks in our study), by accentuating exposure among the most deprived.

Lastly, that the study relies on nitrogen dioxide levels does not mean that we see exposure to this indicator of air quality as a major predictor of pregnancy outcomes. Fine particles PM_2.5,_ and their chemical content, have been shown as hazardous for several pregnancy outcomes [9–14]. Merely, NO_2_, and more generally NOx, are indicators of proximity to emission sources associated with industrial combustion processes, urban heating and petrol or diesel-powered traffic [42]. In the city of Paris, and in general in the Ile-de-France region, wherefrom historical industrial sources have moved to other places in France or abroad, NOx emissions are mainly associated with traffic and secondly with building heating [35]. We computed the correlation between NO_2_ and PM_10_, concentrations for the Paris city monitoring stations during the 2010–2011 periods. This correlation ranged from 0.59 to 0.68 according to the monitoring site (located in the first and 18th arrondissement, respectively), suggesting that our findings for NO_2_ might also be relevant for exposure to PM_10_ and possibly other ambient air pollutants with similar sources. More research is called upon to document this hypothesis.

## Conclusion

In conclusion, our study illustrates that exposure to air pollution during pregnancy may be underestimated when ignoring residential mobility. Underestimation gets stronger as the neighborhood socioeconomic deprivation of the study population increases. However, the spatial pattern of pollution, combined with that of socioeconomic deprivation, might modify this effect, as well as the time of initiation of the pregnancy, following seasonality of ambient air pollutants. Future research conducted in various territories on how different patterns of air pollution and socioeconomic deprivation modify bias in exposure assessment due to geographic mobility is warranted. The findings of such research would provide useful information to identify the population with the highest health risks based on a more accurate individual exposure measure, and hence inspire more effective and targeted actions.

## Additional file

Additional file 1: **Figure S1.** Relation between NO_2_ exposure from the Eq. 1 (the referent model that considers the NO_2_ exposure at the place of residence) and the three other models. **Figure S2.** Paired differences of NO_2_ exposure estimated during the first trimester of pregnancy (in μg/m^3^) between Eqs. 1 and 4 when considering the travel modes associated with different values of NO_2_ concentrations (5th, median and 95th value) extracted from the Montréal study. (DOCX 43 kb)
