# Supplementary material for: Does residential mobility during pregnancy induce exposure misclassification for air pollution?
Source: Environ Health. 2018 Oct 19;17:72. doi: 10.1186/s12940-018-0416-8 (PMC6194718; doi:10.1186/s12940-018-0416-8)
Supplement: Supplementary file 1 — Figure S1. Relation between NO2 exposure from the Eq. 1 (the referent model that considers the NO2 exposure at the place of residence) and the three other models. Figure S2. Paired differences of NO2 exposure estimated during the first trimester of pregnancy (in μg/m3) between Eqs. 1 and 4 when considering the travel modes associated with different values of NO2 concentrations (5th, median and 95th value) extracted from the Montréal study. (DOCX 43 kb) [file 12940_2018_416_MOESM1_ESM.docx]

Figure S1 : Relation between NO_2_ exposure from the model 1 (the referent model that considers the NO_2_ exposure at the place of residence) and the three other models.

Figure S2 : Paired differences of NO_2_ exposure estimated during the first trimester of pregnancy (in μg/m^3^) between models 1 and 4 when considering the travel modes associated with different values of NO_2_ concentrations (5^th^, median and 95^th^ value) extracted from the Montréal study.
